# Prediction of the Rheological Properties of Fresh Cementitious Suspensions Considering Microstructural Parameters

**DOI:** 10.3390/ma15207044

**Published:** 2022-10-11

**Authors:** Sam Rajadurai Rajagopalan, Bang-Yeon Lee, Su-Tae Kang

**Affiliations:** 1 Department of Civil Engineering, Daegu University, 201 Daegudae-ro, Jillyang, Gyeongsan 38453, Korea; 2 School of Architecture, Chonnam National University, 77 Yongbong-ro, Buk-gu, Gwangju 61186, Korea

**Keywords:** rheology, yield stress, plastic viscosity, YODEL, Krieger-Dougherty’s equation

## Abstract

Supplementary cementitious materials (SCMs) are commonly used to partially replace cements. Although it is necessary to investigate the rheological properties of the individual supplementary cementitious materials (SCMs) for understanding complex rheological behaviors of the blended mixes, the study on the investigation of rheological properties of various SCMs such as fly ash, blast-furnace slag, and silica fume, according to various solid volume fractions and prediction models is fairly limited. This study investigated the rheological properties of non-blended cementitious suspensions with Portland cement (PC), fly ash (FA), blast-furnace slag (BS), and silica fume (SF) materials in the experiments and predicted using YODEL (Yield stress mODEL) and Krieger–Dougherty’s (K–D’s) equation. Experiments were designed with various solid volume fractions (ϕ) from 0.28 to 0.44, and the rheological properties of all cementitious suspensions were noted to increase with increasing ϕ, showing an improved flowability at low ϕ. YODEL, derived from the first principles considering particle-size distributions, interparticle forces and microstructural parameters predicted the yield stress. The YODEL predictions were consistent with the experiments with a positive correlation coefficient of above 0.96. K–D’s equation with the maximum particle fractions and intrinsic viscosity as key parameters predicted the plastic viscosity. The K–D’s equation predictions match up with the experiments with a positive correlation coefficient of above 0.94. Both models showed more quantitative predictions without any fitting parameters and could be applied to any multimodal powder suspensions.

## 1. Introduction

The rheological properties of fresh cementitious suspensions are of great interest for many applications in the construction industry [1,2,3,4]. Advances in the construction industry have led to the design of new and special mixtures such as self-compacting, ultra-high-performance, and engineered cementitious composites that can significantly improve the mechanical properties and impermeability, and reduce intrusion of aggressive agents [5,6,7,8,9,10]. These improved characteristics could be hindered by the inadequate rheological properties, which facilitates the placement process such as pumping, casting, and molding [11,12]. Sustainable cementitious mixtures with recycled aggregates or construction waste fines also have difficulty in obtaining proper rheological properties [13,14]. In general, the cementitious mixtures incorporate multi-scale fine particles, admixtures, fibers, superplasticizers, etc., making it difficult to experimentally investigate their rheological properties [15,16]. In addition, there are several factors that influence the rheological properties such as material properties, interparticle forces due to dispersion forces and electrostatic interactions, steric forces from adsorbed polymers, hydrodynamic reactions, and crowding factors [1,2,3,4]. Such complex behaviors make the experimental work more complicated and are in need of adopting multi-scale modeling approaches to predict and understand the rheological properties with minimal experimentations.

Many attempts have received considerable attention in modeling to predict the rheological properties of cementitious suspensions as a function of particle size, interaction forces, and solid volume fractions [17]. There are various models to predict the yield stress of cementitious suspensions considering a range of factors such as volume fraction, water-to-cement ratio, and additives. Legrand proposed a relationship between yield stress and volume fraction (ϕ) in consideration of particle size and shape, and the relationship is applicable when ϕ lies in between 0.475–0.677 [18,19]. Zhou et al. proposed a yield stress model for concentrated flocculated suspensions and investigated the structural impact on yield stress [18]. Zhou et al. model predicted the yield stress of suspensions with ϕ less than 0.42 and suggested that the interparticle forces play a prominent role in determining the structural network strength [20]. Zhou et al. also proposed a general model by considering the particle diameter, Hamaker’s constant, inter-particle distance, and fit parameters [2,20]. Flatt and Bowen proposed a volume fraction-dependent yield stress model for multimodal powder suspensions and named it as YODEL (Yield stress mODEL) [1,2]. YODEL predictions were applied to fit Zhou et al.’s [20] experimental data, and the resultant predictions match up to the experimental results within about 8–9% error rate. [1,2]. Based on the YODEL, Ma et al. proposed a yield stress evolution model that underestimated the experimental results of cement paste with 0.3% nano clay, probably due to the interaction and flocculation between nano clay and ettringite [21]. The effects of specific surfaces and water-to-cement ratio were considered by Lapasin et al. model and noted that the yield stress increases linearly with the increase in specific surfaces [22,23]. Sybertz et al. proposed an equation and studied the substitution effect of fly ash on the rheological behavior of cement paste. The proposed equation provides a good approximation of the measured values [24].

Likewise, there are also various models to predict the plastic viscosity of cementitious suspensions by considering volume fraction, hydration time, and maximum packing fraction. Einstein proposed a simple mathematical expression to evaluate the effect of ϕ on viscosity and was applied to volume fractions less than 5% [25]. An equation was proposed by Mooney for densely packed particles combined with the effect of a crowding factor, which fits the measured experimental data at lower ϕ [26]. Krieger and Dougherty (K–D) proposed a widely applied equation for cementitious suspensions that depends on two parameters, i.e., maximum packing fraction and intrinsic viscosity [27]. The K–D equation was found to provide a good fit across the wide range of ϕ [28]. Chen and Lin combined the K–D equation and established a relationship between the viscosity, volume fraction, and hydration time, indicating that viscosity increases with the hydration time [29]. A two-parameter viscosity equation was proposed by Liu that accurately calculated the maximum packing fraction and predicted the viscosity of various ceramic suspensions more precisely [30].

In the above yield stress and plastic viscosity models, some predictive models have achieved considerable results by adopting microstructural approaches such as particle-size distributions, interparticle forces, and packing fractions. However, the models still have limitations in adoption of fitting parameters and are not applicable to a wide range of ϕ. Among the models discussed above, the most successful model for predicting the yield stress of cementitious suspensions was YODEL, derived from the first principles considering particle-size distributions, interparticle forces and microstructural parameters. In addition, the widely applied equation for plastic viscosity predictions was K–D’s equation because of its simplicity and considered parameters such as the maximum packing fractions and intrinsic viscosity. Both models can be applied to cementitious suspensions with a wide range of ϕ without introducing any fitting parameters. Therefore, the rheological properties of cementitious suspensions were predicted in this study by applying YODEL and the K–D equation in consideration of these advantageous characteristics. It is also noted that previous studies about the YODEL model in cementitious material were limited to the suspensions with cement; thus, information about the parameters of the model applicable for other cementitious particles is hardly found. Meanwhile, K–D equation have been widely applied to general cementitious paste, mortar and concrete, but the applications were undertaken only in macroscopic approach without consideration for the influence of individual material. This shortage limits the extendibility of application of the models to various kinds of cementitious mixtures. Therefore, in this study, it was intended to determine the parameters of the models suitable for suspensions with different kinds of cementitious materials, widening the application.

Moreover, the development of special cementitious mixtures over the last three decades requires a high binder volume, mainly achieved by partially replacing Portland cement (PC) with supplementary cementitious materials (SCMs) [31,32]. SCMs such as fly ash (FA), blast-furnace slag (BS), and silica fume (SF) are commonly used due to their ability to partially replace cement, making a more effective binder [33,34]. In addition to this, SCMs are also used for the purposes of improving strength, particle packing efficiency, and durability, sometimes for the purpose of reducing permeability, alkali-silica reaction, heat evolution during hydration [35,36,37]. To achieve the above intended positive effects, a mixture designed with SCMs should carefully consider the workability as well as the rheological properties. The partial replacement of SCMs can result in complex rheological behavior influenced by the physical properties as well as by the type and replacement ratio. The physical properties including specific surface area (SSA), packing density, particle shape and particle size influence the rheological behavior either by increasing or decreasing the flowability characteristics. Studies were focused on the influence of SCMs to rheology of cement pastes that have been designed as blended mixtures such as binary, tertiary, and quaternary mixtures [38,39,40,41]. However, these complex rheological behaviors are difficult to understand with the blended mixes due to uncontrolled fluctuations in the particle’s properties. This can be achieved by investigating the rheological properties of the individual SCMs as non-blended mixes. Recently, a study designed non-blended cementitious suspensions with individual SCMs that considered the influence of inter-particle distances on the rheological properties [42]. Therefore, more detailed study to investigate the rheological behavior of non-blended mixes could help to better understand each individual rheological behavior and the factors influencing the rheological properties more precisely. For the purpose, this study investigated the rheological properties of non-blended cementitious suspensions with PC, FA, BS, and SF materials in the experiments and predicted using YODEL (Yield stress mODEL) and Krieger–Dougherty’s (K–D’s) equation. Based on the rheological information of non-blended mixes, the rheological properties of any blended mixtures with SCMs can be predicted and controlled to obtain more effective mixtures with adequate rheological properties.

## 2. Yield Stress and Plastic Viscosity Models: Scientific Background

### 2.1. YODEL

Flatt and Bowen [1,2] proposed YODEL, a yield stress model for multimodal suspensions based on the microstructural considerations of colloidal particle interactions. This equation was derived in consideration of the interparticle forces, suspension microstructure, and particle-size distribution. This also includes the physical parameters such as particle size, geometrical maximum packing fraction, percolation threshold, and minimum separation distance at contact. Based on the YODEL, the yield stress function ($\tau_{0}$) was expressed as in Equation (1),

(1) $$\tau_{0}=m_{1}\frac{\phi {\left(\phi -\phi_{0}\right)}^{2}}{\phi_{m}\left(\phi_{m}-\phi \right)}$$

where, $m_{1}$ indicates the predetermined factor that considers interparticle forces, particle size, and particle-size distribution as expressed in Equation (2). ϕ represents the solid volume fraction of the cementitious suspensions. $\phi_{0}$ represents the percolation volume fraction that depends on the interaction between Brownian motion (dispersive) and colloidal attractive forces between particles. $\phi_{m}$ represents the maximum packing fraction of cementitious particles.

(2) $$m_{1}=\frac{1.8}{\pi^{4}}G_{max}a^{*}u_{k,k}\left(\frac{f_{\sigma ,\Delta }^{*}}{R_{v,50}^{2}}\right)$$

where, $R_{v,50}$ was the median volume radius, $\frac{f_{\sigma ,\Delta }^{*}}{R_{v,50}^{2}}$ was derived from the particle-size distribution, and $a^{*}$ indicates the average (characteristic) radius of curvature at particle contacts, which was introduced to describe the dependence of *m*_1_ on interparticle force. $G_{max}$ was the maximum attractive interparticle force normalized by the radius of curvature at the contact points as expressed in Equation (3),

(3) $$G_{max}≅\frac{A_{0}}{12H^{2}}$$

where, $A_{0}$ and H represents the Hamaker’s constant and minimum separation distance. In addition, the effect of particle-size distribution was considered by the function $f_{\sigma ,\Delta }^{*}$ and expressed in Equation (4),

(4) $$f_{\sigma ,\Delta }^{*}=\frac{1}{u_{k,k}}\overset{m}{\underset{k=1}{\sum }}\phi_{k}\;\overset{m}{\underset{l=1}{\sum }}S_{a,1}\frac{A_{s}}{A_{c}}\frac{\Delta v_{k,1}}{b_{k}^{3}}\frac{1}{\left(b_{k}^{2}-b_{1}^{2}\right)}$$

where, $b_{i}$ is the normalized particle radii by normalizing the particle radii $a_{i}$ by the mean volume radius $R_{v,50}$. $\phi_{k}$ indicates the volume fraction of particles of size $b_{k}$ in the size interval k. $\frac{A_{s}}{A_{c}}$ and $S_{a,1}$ were derived from the geometrical model of Suzuki et al. [43], as shown in Equations (5) and (6). $\Delta v_{k,1}$ was a geometrical term that accounts for a change in the maximum packing fraction induced by each pair of undispersed particles of sizes $a_{k}$ and $a_{l}$, which is expressed as shown in Equation (7). $u_{k,k}$ indicates the normalized factor as expressed in Equation (8), with the assumption of enclosing sphere model for the effective volume fraction of solids. In Flatt and Bowen [1,2], three geometrical models (truncated cone model without the particle portion, truncated cone model with the particle volume, and the enclosing sphere model without the particle volume) for effective volume fraction of solids were introduced to account for an increase in the effective volume of solids from undispersed particles, which is used in calculating $\Delta v_{k,1}$ and $u_{k,k}$. Given that explaining the choice of a geometrical model is somewhat arbitrary, they suggested that the enclosing sphere model provided a better result in the data analysis, even though the impact of this choice has a less pronounced effect on size distribution. Therefore the equations of $\Delta v_{k,1}$ and $u_{k,k}$ based on the enclosing sphere model were adopted for this study.

(5) $$\frac{A_{s}}{A_{c}}=\frac{2\left(b_{1}+b_{k}\right)}{b_{k}+b_{1}-\sqrt{b_{k}\left(b_{k}+2b_{1}\right)}}$$

(6) $$S_{a,1}=\frac{\emptyset_{l}/b_{1}}{\sum_{i=1}^{m}\emptyset_{i}/b_{i}}$$

(7) $$\Delta v_{k,1}=4\pi \left(b_{k}b_{1}\right)\left(b_{k}+b_{1}\right)$$

(8) $$u_{k,k}=\frac{16\pi }{2-\sqrt{3}}$$

### 2.2. Krieger–Dougherty’s (K–D) Equation

The K–D’s equation [27] predicts the plastic viscosity from the volume fraction of cementitious particles as expressed in Equation (9),

(9) $$\mu_{0}\left(1-\frac{\phi }{\phi_{m}}\right){\;}^{-\left[\eta \right]\phi_{m}}$$

where, μ represents the plastic viscosity of the suspensions; $\mu_{0}$ represents the plastic viscosity of the fluid phase of the suspensions; ϕ indicates the solid volume fraction of the cementitious suspensions; $\phi_{m}$ indicates the maximum packing fraction of cementitious particles, and; [η] represents the intrinsic viscosity which mainly depends on the shape of particles.

## 3. Experimental Program

### 3.1. Materials

**Figure 1:**
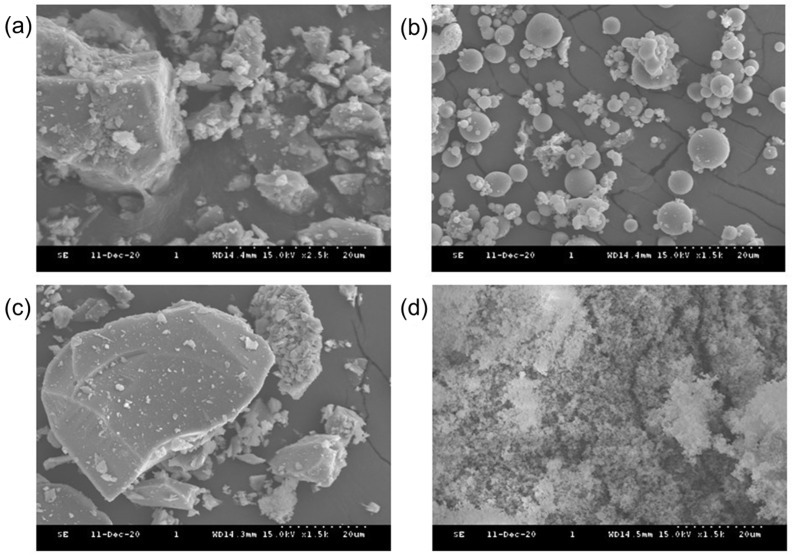
SEM micrographs of cementitious materials: (**a**) PC; (**b**) FA; (**c**) BS; (**d**) SF.

**Figure 2:**
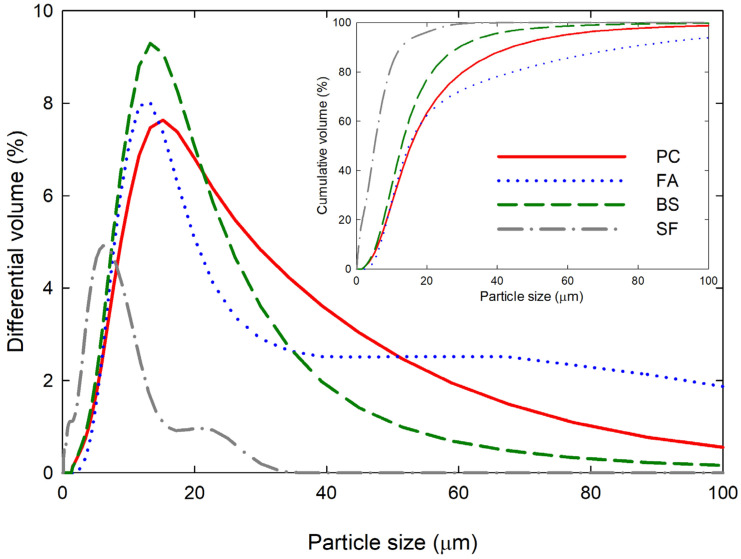
Particle-size distributions (PSDs) of cementitious materials.

The raw materials used in this study consisted of: PC, FA, BS, and SF materials. The physical properties and the mean particle sizes (D_mean_) of the PC, FA, BS, and SF are shown in Table 1. A scanning electron microscope (SEM, S-4300, Hitachi, Tokyo, Japan) was used to observe the micro-morphology of cementitious materials as shown in Figure 1. In the SEM images, the PC and BS consist of mostly angular particles, FA consists of spherical particles, and SF consists of an agglomerated spheroidal particle. The chemical compositions of PC, FA, BS, and SF were obtained using an X-ray fluorescence spectrometer (PW2400, Philips, Amsterdam, The Netherland), of which the main oxides are presented in Table 2. The cementitious materials predominantly consist of major oxides that includes SiO_2_, Al_2_O_3_, CaO, and Fe_2_O_3_, and a small amount of minor oxide such as MgO, respectively. A laser scattering particle-size distribution analyzer (LS 13 320, Beckman Coulter, Brea, CA, USA) was used to measure the particle-size distributions (PSDs) of PC, FA, BS, and SF particles as shown in Figure 2.

### 3.2. Mixture Formulations and Procedures

Four groups of non-blended mixtures were prepared using PC, FA, BS, and SF materials with different water-to-solid volume ratios. The water-to-solid volume ratios were quantified as the volumetric ratios, as the rheological properties generally governed by volume ratio rather than weight ratio. All the mixtures were proportioned with different water-to-solid volume ratios (w/s)_v_ varied from 1.25, 1.50, 1.75, 2.00, 2.25, to 2.50, which correspond to the mass-based water-to-solid volume ratios (w/s)_m_ between 0.40 to 1.00. The mix ratios that includes (w/s)_v_, and the corresponding (w/s)_m_ ratios, and ϕ are shown in Table 3. For an individual cementitious material, six non-blended mixtures were prepared with different (w/s)_v_ ratios, which totally consist of twenty-four mixtures. All the materials were preconditioned to a constant temperature (20 ± 3 °C) for 24 h inside the laboratory to minimize the temperature difference. To produce the mixtures, the solid ingredients were mixed in a dry state for 2 min in a Hobart-mixer. The water was slowly added to the dry mixture and mixed for about 3 min. Then, the sides of the mixer container were scraped, and the mixing continued until a homogeneous mixture was obtained.

### 3.3. Rheological Measurements

**Figure 3:**
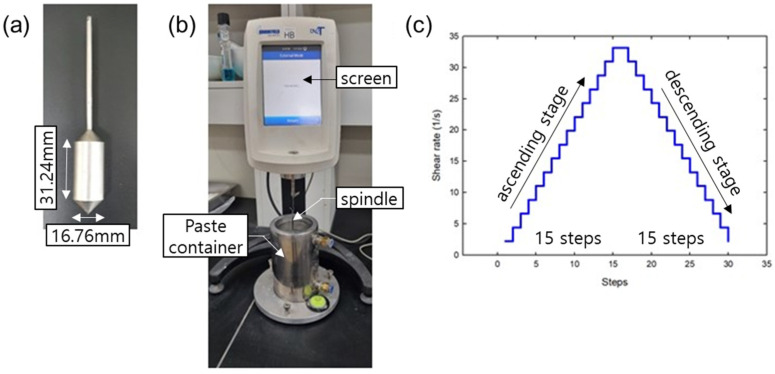
(**a**) Spindle type; (**b**) Experimental set-up; (**c**) Shear rate protocol applied for experiments.

Immediately after the mixing, the mixtures were subjected to rheological tests using a Brookfield DV2T rheometer(AMETEC Brookfield, Middleborough, MA, USA) equipped with mortar type spindle. The rheometer measured the shear stress response to the applied strain rate, and the data was recorded using a PC connected to the rheometer. Figure 3 shows the spindle type, experimental set-up, and applied shear rates. This study adopted the Bingham model, which includes yield stress and viscosity as shown in Equation (10),

(10) $$\tau =\tau_{0}+\mu \gamma$$

where, $\tau_{0}$ and μ indicate the yield stress and plastic viscosity of the cementitious suspensions. The torque required to rotate the spindle in the mixtures was determined, and the shear rate was increased from 0 to 33.15 s^−1^ and decreased from 33.15 to 0 s^−1^. The shear rate at each step was maintained for 10 s to detect a stable shear stress. The curves at the decreasing rate were more consistent and a linear regression was performed to determine the plastic viscosity and yield stress with the slope and intercept of the regression analysis line plotted through shear stress against shear rate. For each mixture sample, three repetitive tests were performed, and the average yield stress and plastic viscosity were obtained.

## 4. Results and Discussions

### 4.1. Yield Stress of the Cementitious Suspensions

#### 4.1.1. Yield Stress in the Experiments with Different ϕ

**Figure 4:**
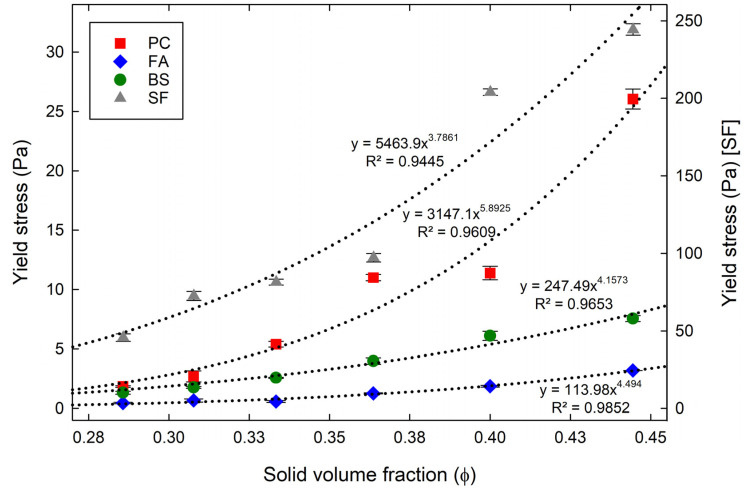
Yield stress of cementitious suspensions with different ϕ in the experiments.

From the rheological tests, the yield stresses of cementitious suspensions were obtained using a linear regression plotted through shear stress on the shear rate. Three tests were performed for each mixture and the average values with deviations were also obtained. Figure 4 shows the obtained yield stress of cementitious suspensions with different ϕ. For all the cementitious suspensions, the yield stresses were noted to increase with increasing ϕ, showing an improved flowability with the low ϕ. A non-linear relationship between ϕ and yield stress was established using the relationship $y=ax^{b}$, as shown in the figure. By using the relationship, the maximum yield stress (a), which can be theoretically obtained at ϕ = 1 and the increase factor (b) with increasing ϕ (x) were estimated. The maximum yield stresses of the PC, FA, BS, and SF suspensions were 3147.1 Pa, 113.98 Pa, 247.49 Pa, and 5463.9 Pa, respectively. The increase factors with increasing ϕ of the PC, FA, BS, and SF suspensions were 5.90, 4.50, 4.16, and 3.79, respectively. The above relationship can be used to predict the yield stress of the cementitious suspensions with different ϕ. Moreover, when comparing the yield stress of the PC with the FA and BS suspensions at the same ϕ, the FA and BS suspensions were observed to provide low yield-stress values, while the SF suspensions showed the opposite tendency. The yield stress of the FA suspensions reduced by 75% to 90% compared to PC suspensions, indicating that the FA suspensions were more fluidic mixtures. These fluidic observations can be governed by the spherical shape of FA particles and the filling effects that increase the physical separation between particles, which reduces the yield stress [44,45]. Similar to the FA suspensions, the BS suspensions have lower yield-stress values than the PC suspensions, i.e., reduced by 28% to 70%. This behavior may be associated with the smooth surface of BS particles, low-chemical effect, and the micro-filling effect [38,39]. In contrast, the yield stress of the SF suspensions increased significantly by 782% to 2372% compared to the PC suspensions. This can be explained by the dense packing and the fine size of SF particles, which results in the reduction in particle spacing and an increase in the number of direct contact points between particles, thus making it difficult for the particles to slide on each other [add]. The increase in thixotropy with SF is also unfavorable to the initial flow [35]. In addition, SF suspension shows relatively higher variation from the regression curve. It seems to be due to the difficulty in obtaining good dispersion of the fine particles with consistency because of the higher possibility of agglomeration for finer particles. The same tendency can be found in the results of plastic viscosity.

#### 4.1.2. Calculation of YODEL Parameters

**Figure 5:**
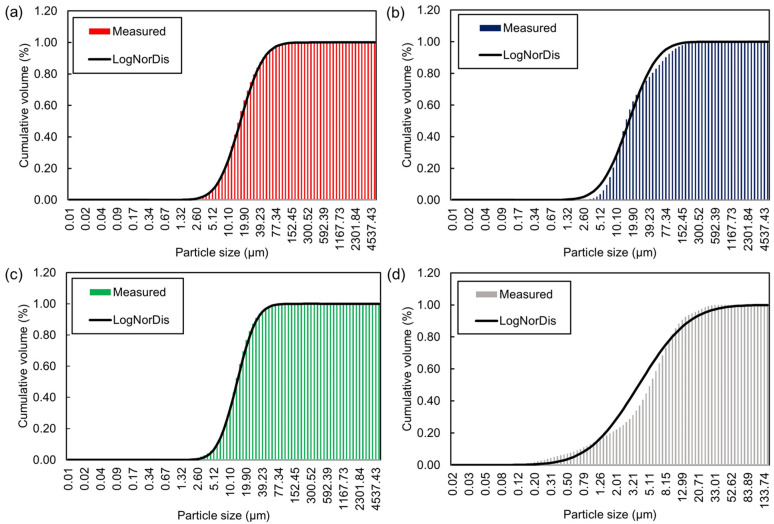
Log-normal distributions of the cementitious materials: (**a**) PC; (**b**) FA; (**c**) BS; (**d**) SF.

To obtain quantitative predictions using YODEL, the first step was to measure the PSDs of the cementitious materials. The PSDs of the cementitious materials were measured using the laser scattering particle-size distribution analyzer and are displayed in Figure 2. From the PSDs, the mean particle sizes of the PC, FA, BS, and SF were 21.58 µm, 30.20 µm, 15.85 µm, and 6.31 µm, respectively. In general, the cementitious materials exhibit a quasi-log-normal size distribution, and the cumulative distribution must fit well with the log-normal distributions, so that the $f_{\sigma ,\Delta }^{*}$ values can be estimated using directly measured PSDs [1,2]. The log-normal size distributions of the cementitious materials were obtained and shown in Figure 5. The obtained log-normal distributions were fitted well to the cumulative distributions for all the cementitious materials. Therefore, the $f_{\sigma }^{*}$ values were estimated as a function of the normal PSDs. The mean and median particle sizes of the cementitious materials, and the estimated $f_{\sigma ,\Delta }^{*}$ and $u_{k,k}$ (calculated using enclosing sphere model) values are shown in Table 4. The $G_{max}$ values were then calculated using $A_{0}$ and H. Firstly, $A_{0}$ were estimated by using Equation (11) for the cementitious particles of same kind,

(11) $$A_{0}={\left(\sqrt{A_{1}}-\sqrt{A_{3}}\right)}^{2}$$

where, $A_{1}$ and $A_{3}$ indicates the Hamaker’s constant of cementitious particles and the suspending fluid (Water = 4.38 × 10^−20^ J). The $A_{1}$ was estimated by using the rule of mixtures as shown in Equation (12),

(12) A1=m1m×Ah1+m2m×Ah2+⋯+mnm×Ahn

where, $m_{1}$, $m_{2}$, and $m_{n}$ indicates the mass (%) of each chemical component of cementitious materials; m indicates the total mass (%) of cementitious materials; $Ah_{1}$, $Ah_{2}$, and $Ah_{n}$ represents the corresponding Hamaker’s constant of each chemical component. The chemical components of the cementitious materials measured through X-ray fluorescence spectroscopy and their corresponding Hamaker’s constants [46,47] are given in Table 5. As shown in the table, the estimated $A_{0}$ of the PC, FA, BS, and SF suspensions were 1.628 × 10^−20^ J, 1.449 × 10^−20^ J, 1.638 × 10^−20^ J, and 6.885 × 10^−21^ J, respectively. In the cementitious suspensions, the H lies in the order of 2 nm for flocculated systems and 10 nm for fully dispersed systems [4]. This study investigated the rheological properties of cementitious suspensions without any chemical additives such as superplasticizers, and therefore the suspensions were not totally dispersed systems. The H values were adjusted to fit the experimental yield-stress values and the obtained H of the PC, FA, BS, and SF suspensions were 2.5 nm, 7.0 nm, 6.1 nm, and 0.5 nm, respectively. Then, $m_{1}$ depending on the interparticle forces was calculated, assuming the contact points between particles as the average fixed radius of curvature $a^{*}$ [1,2]. The interparticle forces would not be proportional to the particle size of a material, but largely independent of the particle size and $a^{*}$ is therefore determined for each material regardless of particle size. The $a^{*}$ of alumina particles was estimated as 20 nm in Flatt and Bowen [1,2], and Roussel et al. [4] suggested that the $a^{*}$ of cement particles was 20 times larger than alumina particles. Considering the lack of informative previous studies about $a^{*}$ for all the materials and the limited experiments for this study, a constant $a^{*}$ was assumed for all the cementitious materials used for simplicity of analysis. Therefore, the $a^{*}$ was fixed as 300 nm for all the cementitious particles. The $\phi_{m}$ was calculated using de Larrard’s compressive packing model (CPM) [48], and the calculated $\phi_{m}$ of the PC, FA, BS, and SF particles were 0.59, 0.60, 0.59, and 0.69, respectively. In addition, the $\phi_{0}$ values considered for the cementitious materials were ranged from 0.024 to 0.04 and was assumed as 0.03 for all the suspensions [4]. The $a^{*}$, $\phi_{0}$, and $\phi_{m}$ are given in Table 6.

#### 4.1.3. Prediction of Yield Stress Using YODEL

**Figure 6:**
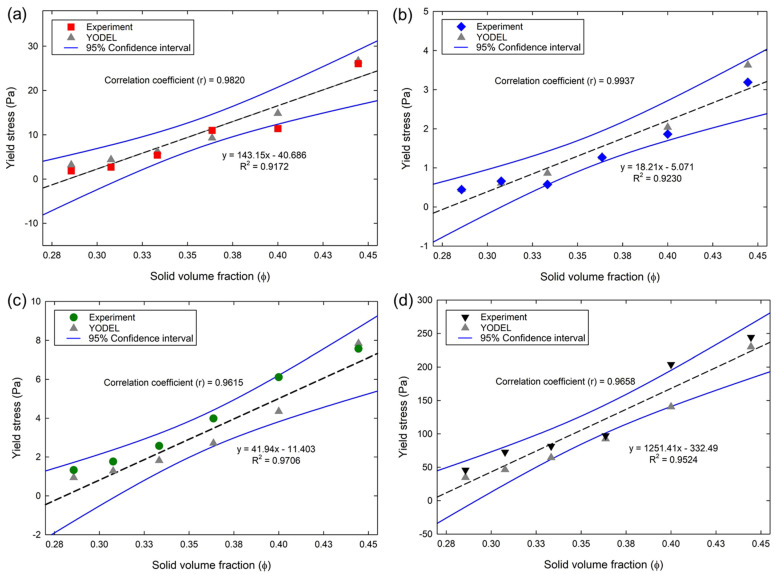
Yield stress of cementitious suspensions in YODEL predictions and experiments: (**a**) PC; (**b**) FA; (**c**) BS; (**d**) SF.

By using the above estimated microstructural parameters required by YODEL, the yield stresses of the cementitious suspensions were predicted and fitted with the experimental data. Figure 6 shows the yield stress of cementitious suspensions obtained through the experiments and predicted through YODEL. Similar to the experimental data, the yield stresses of all the cementitious suspensions increased with the increase in ϕ in the YODEL predictions. The correlation coefficient (*r*), and the regression analysis with 95% confidence intervals (i.e., maximum, and minimum) were estimated between the experiments and YODEL predictions, as shown in the figure. In the PC suspensions, the YODEL predicted the yield stress with a mean standard deviation to the experimental data of approximately 1.14. In addition, the *r* between the experiment and prediction was 0.98, respectively. The average standard deviation and *r* values between the experiment and prediction of FA suspensions were 0.11 and 0.99, respectively. For the BS suspensions, the average standard deviation and *r* values between the experiment and prediction were 0.58 and 0.96, respectively. In addition, the average standard deviation and *r* values between the experiment and prediction of SF suspensions were 16 and 0.96, respectively. From the above results, the YODEL predicted the yield stress of cementitious suspensions with positive *r* above 0.96. As YODEL adopts microstructural approaches to predict yield stress as a function of PSDs, interparticle forces, and packing fractions, a more quantitative predictive capacity becomes apparent and was certainly confirmed in the above results. The PSDs plays a very prominent role, as it is the main input parameter. The YODEL also designed without introducing any fitting parameters and introduced CPM model to estimate $\phi_{m}$. Therefore, an appropriate input of PSDs and $\phi_{m}$ was very essential for YODEL to obtain quantitative predictions.

### 4.2. Plastic Viscosity of the Cementitious Suspensions

#### 4.2.1. Plastic Viscosity in the Experiments with Different ϕ

**Figure 7:**
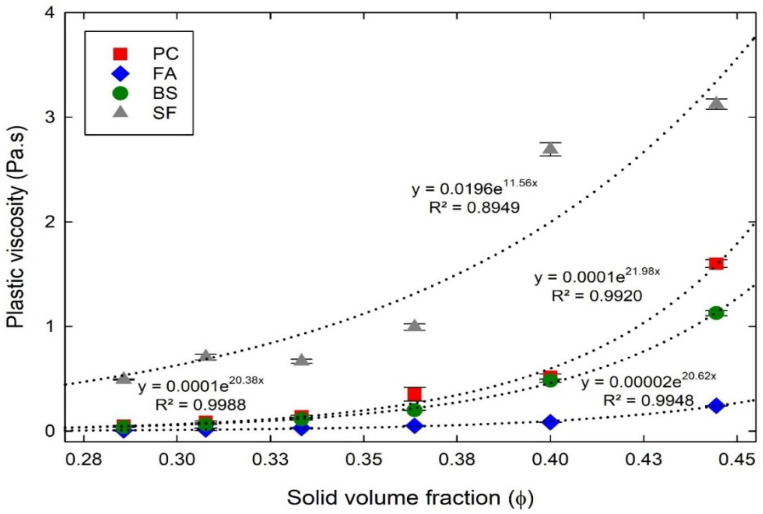
Plastic viscosity of cementitious suspensions with different ϕ in the experiments.

Figure 7 shows the obtained plastic viscosity of cementitious suspensions with different ϕ. For all the cementitious suspensions, the plastic viscosity were noted to increase with increasing ϕ, showing an improved flowability with the low ϕ. A non-linear relationship between ϕ and plastic viscosity was established using the exponential function ($y=ae^{-bx}$). By using the relationship, the initial plastic viscosity (a) when the ϕ was 0, and the increase factor (b) with increasing ϕ was estimated. By using the relationship, the plastic viscosity of cementitious suspensions with different ϕ can be predicted. The initial plastic viscosity was the measure of water, which means that there was no solid particles. The initial plastic viscosity of the PC, FA, BS, and SF suspensions were predicted as 0.0001 Pa.s, 0.00002 Pa.s, 0.0001 Pa.s, and 0.0196 Pa.s, which were relatively equivalent to those of water (0.01 Pa.s at 20 °C, decreases as the temperature increases) [49] respectively. The low initial plastic viscosity of cementitious suspensions predicted from the experimental results may be due to the chemical reactions and initial hydration process that immediately raises the temperature when mixed with water. The increase factor with increasing ϕ of the PC, FA, BS, and SF suspensions were 21.98, 20.62, 20.38, and 11.56, respectively. Similar to the yield stress, the FA and BS suspensions were observed to provide lower plastic viscosity values than PC suspensions, whereas an opposite behavior was observed in SF suspensions. The plastic viscosity of the FA suspensions reduced by 78% to 86% compared to PC suspensions, indicating that the FA suspensions were more fluidic mixtures. These fluidic observations were explained above due to shape and filling effects [44,45]. The BS suspensions also showed a lower plastic viscosity value than the PC suspensions, reduced by 8% to 44%. It was due to the smooth surface of BS particles, low-chemical and micro-filling effects [38,39]. On contrary, the plastic viscosity of the SF suspensions increased significantly by 95% to 830% compared to the PC suspensions, caused by the fine size and dense packing. As explained previously, those properties make it difficult for the particles to slide over each other because of increased contacts between particles. SF suspension therefore presented much higher viscosity than PC, FA, and BS suspensions with a constant solid volume fraction.

#### 4.2.2. Calculation of K–D’s Equation Parameters

The K–D equation mainly depends on two parameters: $\phi_{m}$ and [η]. The $\phi_{m}$ for all the cementitious materials were calculated using de Larrard’s CPM model [48]. The calculated $\phi_{m}$ of PC, FA, BS, and SF particles were 0.59, 0.60, 0.59, and 0.69, respectively. When the $\phi_{m}$ ranging between 0.6–0.7, the [η] was assumed as 2.5 for monodisperse and polydisperse systems, 3 to 5 when the particles were sharp and angular, and between 4 to 10 when the particles consist of acicular, rods and fiber shapes [50]. The [η] depends on the individual effect of particles and their shape [28,50,51,52]. The [η] can be chosen as 2.5 for rigid spherical geometries and must be modified if the particle shapes were found to be deviated [50,51,52]. An expression suggested by Pabst et al. [53] can be useful to estimate [η], but the correlation between particle shape and [η] was fundamentally complicated [50,51,52]. However, [η] value generally assumes that all particles have a similar shape and most of the studies adopted [η] to be adjusted to fit the experimental measurements [50,51,52]. Therefore, this study also adopted the [η] to fit the K–D’s equation by adjusting the values to fit the experimental results. The adopted [η] values of PC, FA, BS, and SF particles were 9.0, 7.0, 8.5, and 13.0, respectively. Table 7 shows the estimated $\phi_{m}$ and [η].

#### 4.2.3. Prediction of Plastic Viscosity Using K–D’s Equation

**Figure 8:**
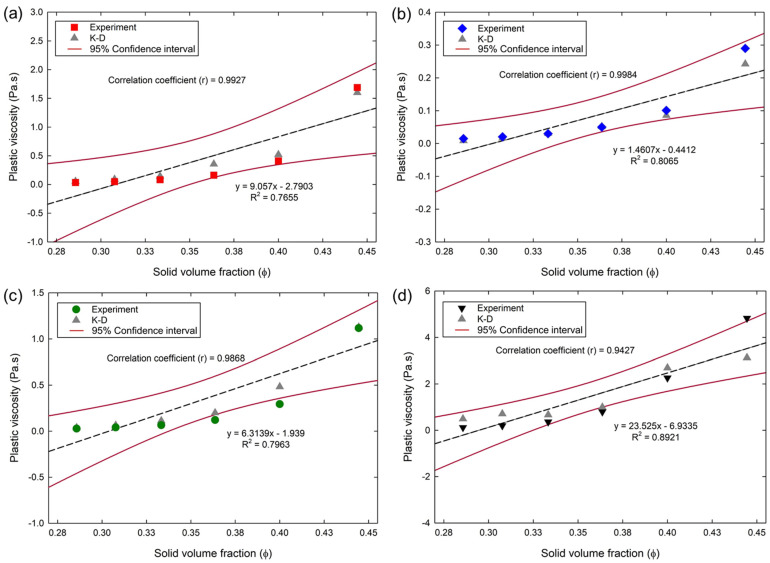
Plastic viscosity of cementitious suspensions in K–D predictions and experiments: (**a**) PC; (**b**) FA; (**c**) BS; (**d**) SF.

From the adopted K–D’s equation parameters, the plastic viscosities of the cementitious suspensions were predicted and depicted in Figure 8. The K–D’s equation also predicted the plastic viscosities of cementitious suspensions with a nominal error rate. The plastic viscosities of all the cementitious suspensions increased with the increase in ϕ in the K–D’s predictions, which was the similar behavior obtained in the experiments. The K–D’s equation predicted the plastic viscosity of the PC suspensions with an average standard deviation and *r* to the experiments of about 0.059 and 0.99, respectively. The average standard deviation and *r* between the experiment and prediction of the FA suspensions were 0.008 and 0.99, respectively. For the BS suspensions, the average standard deviation and *r* were 0.042 and 0.98, respectively. In addition, the average standard deviation and *r* between the experiment and prediction of SF suspensions were 0.41 and 0.94, respectively. Low deviations and high *r* values in the analysis indicates a good approximation by K–D’s equation for all the suspensions. As the K–D’s equation depends on $\phi_{m}$ and [η], the CPM model was found to be remarkably effective in providing a good prediction, which resulted in the nominal deviations and positive *r* of above 0.94 for all the suspensions.

## 5. Conclusions

This study primarily investigated the rheological properties of cementitious suspensions, designed as non-blended mixtures with different ϕ. Based on the experimental and analytical results, the following conclusions can be drawn:

(1) In the experiments, the rheological properties of all the cementitious suspensions were noted to increase with increasing ϕ, showing an improved flowability with the low ϕ. Compared to the PC suspensions, the FA and BS suspensions showed an improved flowability, which was mainly due to the particle characteristics. The shape of FA was spherical, which facilitates isolation and dispersion, thus providing more flowable mixtures. In the case of BS, the improved flowability can be due to its smooth surface, less-chemical activity, micro-filling effect, and large surface area. However, the SF suspensions showed an opposite behavior, which was less flowable. As the SF consists of more fine particles, it could reduce particle spacing and increases direct contact points between particles, which makes it difficult for the particles to slide each other.
(2) The yield stress of all the cementitious suspensions increased with the increase in ϕ in the YODEL predictions. Using the YODEL, the yield stresses of the PC, FA, BS, and SF suspensions were predicted with positive *r* to the experiments of approximately 0.98, 0.99, 0.96, and 0.96, respectively. An appropriate input of PSDs and $\phi_{m}$ without introducing any additional fitting parameters, the YODEL predictions consistent with the experiments with positive correlations. The YODEL can be applied to all multimodal powder suspensions, which provides a more quantitative predictive capacity compared to other models, as it was derived based on the microstructural parameters and more related to the true physical properties.
(3) The plastic viscosities of the PC, FA, BS, and SF suspensions were predicted with the correlation coefficient between K–D’s and experiment of approximately 0.99, 0.98, 0.98, and 0.94, respectively. As the K–D’s equation depends on $\phi_{m}$ and [η] , a proper estimation of $\phi_{m}$ and [η] results in a good prediction of plastic viscosities and can be applied to different multimodal and complex powder suspensions.
(4) The YODEL and K–D’s equation provided more consistent results for the non-blended cementitious mixtures with minimum deviations and positive correlations, and these models will be applied to blended (i.e., binary, tertiary, and quaternary) cementitious mixtures in the future studies.

## Figures and Tables

**Table 1:** Physical properties of cementitious materials.

Materials	Density (g/cm^3^)	Specific Surface Area SSA (cm^2^/g)	Mean Size (D_mean_) (µm)
PC	3.15	2800	21.58
FA	2.23	3860	30.20
BS	2.90	4530	15.85
SF	2.30	150,000	6.31

**Table 2:** Chemical compositions of cementitious materials.

Components	PC (%)	FA (%)	BS (%)	SF (%)
SiO_2_	20.50	53.70	33.80	99.10
Al_2_O_3_	5.11	25.70	13.90	-
CaO	62.00	-	44.20	-
MgO	2.60	-	3.57	-
Fe_2_O_3_	3.30	5.76	-	-

**Table 3:** Mix ratios of the cementitious suspensions.

Water-to-Solid Volume Ratios (w/s)_v_	Solid Volume Fraction (ϕ)	Mass-Based Water-to-Solid Volume Ratios (w/s)_m_			
		PC	FA	BS	SF
1.25	0.44	0.39	0.56	0.43	0.54
1.50	0.40	0.47	0.67	0.51	0.65
1.75	0.36	0.55	0.78	0.60	0.76
2.00	0.33	0.63	0.89	0.68	0.86
2.25	0.30	0.71	1.00	0.77	0.97
2.50	0.28	0.79	1.12	0.86	1.08

**Table 4:** Calculated particle size parameters for the YODEL.

Materials	Mean Diameter $D_{90}$ (µm)	Median Diameter $D_{50}$ (µm)	Median Radius $R_{v,50}$ (µm)	$u_{k,k}$	$f_{\sigma ,\Delta }^{*}$
PC	21.58	15.44	7.71	187.594	861.66
FA	30.20	14.91	7.45		1007.43
BS	15.85	12.81	6.41		1001.71
SF	6.31	5.14	2.57		148.24

**Table 5:** Chemical compositions and their Hamaker’s constant of cementitious materials [46,47].

Compositions	Hamaker’s Constant (× 10^−20^ J)	PC (%)	FA (%)	BS (%)	SF (%)
SiO_2_	8.53	20.50	53.70	33.80	99.10
Al_2_O_3_	15.5	05.11	25.70	13.90	-
CaO	12.4	62.00	-	44.20	-
MgO	10.6	02.60	-	03.57	-
Fe_2_O_3_	25.2	03.30	05.76	-	-
Hamaker’s constant (A_1_, ×10^−20^ J)	-	11.33	10.01	11.41	8.53

**Table 6:** Estimated radius of curvature ($a^{*}$ ), percolation volume fraction ($\phi_{0}$), and maximum solid volume fraction ($\phi_{m}$).

Materials	$\mathrm{Radius}\;\mathrm{of}\;\mathrm{Curvature}\;(a^{*})$	$\mathrm{Percolation}\;\mathrm{Volume}\;\mathrm{Fraction}\;(\phi_{0})$	$\mathrm{Maximum}\;\mathrm{Solid}\;\mathrm{Volume}\;\mathrm{Fraction}\;(\phi_{m})$
PC	300 nm	0.03	0.59
FA			0.60
BS			0.59
SF			0.69

**Table 7:** Maximum solid volume fraction ($\phi_{m}$) and intrinsic viscosity ([η]).

Materials	$\mathrm{Maximum}\;\mathrm{Solid}\;\mathrm{Volume}\;\mathrm{Fraction}\;(\phi_{m})$	Intrinsic Viscosity ([η])
PC	0.59	9.0
FA	0.60	7.0
BS	0.59	8.5
SF	0.69	13.0

## Data Availability

Not applicable.

## References

[1] Flatt R.J., Bowen P. (2006). Yodel: A yield stress model for suspensions. J. Am. Ceram. Soc..

[2] Flatt R.J., Bowen P. (2007). Yield stress of multimodal powder suspensions: An extension of the YODEL (Yield Stress mODEL). J. Am. Ceram. Soc..

[3] Flatt R.J. (2004). Towards a prediction of superplasticized concrete rheology. Mater. Struct..

[4] Roussel N., Lemaître A., Flatt R.J., Coussot P. (2010). Steady state flow of cement suspensions: A micromechanical state of the art. Cem. Concr. Res..

[5] Roussel N., Bessaies-Bey H., Kawashima S., Marchon D., Vasilic K., Wolfs R. (2019). Recent advances on yield stress and elasticity of fresh cement-based materials. Cem. Concr. Res..

[6] Li Y., Li J., Yang E.-H., Guan X. (2022). Mechanism study of crack propagation in river sand engineered cementitious composites (ECC). Cem. Concr. Compos..

[7] Pang L., Liu Z., Wang D., An M. (2022). Review on the Application of Supplementary Cementitious Materials in Self-Compacting Concrete. Crystals.

[8] Kang S.T., Kim J.K. (2011). The relation between fiber orientation and tensile behavior in an ultra high performance fiber reinforced cementitious composites (UHPFRCC). Cem. Concr. Res..

[9] Kang S.T., Lee Y., Park Y.D., Kim J.K. (2010). Tensile fracture properties of an ultra high Performance Fiber Reinforced Concrete (UHPFRC) with steel fiber. Comput. Struct..

[10] Lee Y., Kang S.T., Kim J.K. (2010). Pullout behavior of inclined steel fiber in an ultra-high strength cementitious matrix. Constr. Build. Mater..

[11] Yoo D.-Y., Yoon Y.-S. (2016). A review on structural behavior, design, and application of ultra-high-performance fiber-reinforced concrete. Int. J. Concr. Struct. Mater..

[12] Soliman N., Tagnit-Hamou A. (2016). Development of ultra-high-performance concrete using glass powder—Towards ecofriendly concrete. Constr. Build. Mater..

[13] Ma Z., Shen J., Wang C., Wu H. (2022). Characterization of sustainable mortar containing high-quality recycled manufactured sand crushed from recycled coarse aggregate. Cem. Concr. Compos..

[14] Liu M., Wu H., Yao P., Wang C., Ma Z. (2022). Microstructure and macro properties of sustainable alkali-activated fly ash mortar with various construction waste fines as binder replacement up to 100%. Cem. Concr. Compos..

[15] Meng W., Khayat K.H. (2016). Mechanical properties of ultra-high-performance concrete enhanced with graphite nanoplatelets and carbon nanofibers. Compos. Part B.

[16] Huang H., Gao X., Jia D. (2019). Effects of rheological performance, antifoaming admixture, and mixing procedure on air bubbles and strength of UHPC. J. Mater. Civ. Eng..

[17] Banfill P.F. The rheology of fresh cement and concrete—A review. Proceedings of the 11th International Cement Chemistry Congress.

[18] Legrand C. (1970). Rheologie des melanges de ciment ou de sable et d’eau. Rev. Mater. Constr. Trav. Publics Cim. Beton.

[19] Tao C., Kutchko B.G., Rosenbaum E., Massoudi M. (2020). A review of rheological modeling of cement slurry in oil well applications. Energies.

[20] Zhou Z., Solomon M.J., Scales P.J., Boger D.V. (1999). The yield stress of concentrated flocculated suspensions of size distributed particles. J. Rheol..

[21] Ma S., Kawashima S. (2019). A rheological approach to study the early-age hydration of oil well cement: Effect of temperature, pressure and nanoclay. Constr. Build. Mater..

[22] Lapasin R., Papo A., Rajgelj S. (1983). The phenomenological description of the thixotropic behaviour of fresh cement pastes. Rheol. Acta.

[23] Lapasin R., Longo V., Rajgelj S. (1979). Thixotropic behaviour of cement pastes. Cem. Concr. Res..

[24] Sybertz F., Reick P. (1991). Effect of fly as on the rheological properties of cement paste. Rheol. Fresh Cem. Concr..

[25] Einstein A. (1906). Eine neue bestimmung der moleküldimensionen. Ann. Phys..

[26] Mooney M. (1951). The viscosity of a concentrated suspension of spherical particles. J. Colloid Sci..

[27] Krieger I.M., Dougherty T.J. (1959). A mechanism for non-newtonian flow in suspensions of rigid spheres. Trans. Soc. Rheol..

[28] Justnes H., Vikan H. (2005). Viscosity of cement slurries as a function of solids content. Ann. Trans. Nordic Rheol. Soc..

[29] Chen C.-T., Lin C.-W. (2017). Stiffening behaviors of cement pastes measured by a vibrational viscometer. Adv. Civ. Eng. Mater..

[30] Liu D.-M. (2000). Particle packing and rheological property of highly-concentrated ceramic suspensions: φ_m_ determination and viscosity prediction. J. Mater. Sci..

[31] Kuzielová E., Žemlička M., Novotný R., Palou M.T. (2018). Simultaneous effect of silica fume, metakaolin and ground granulated blast-furnace slag on the hydration of multicomponent cementitious binders. J. Therm. Anal. Calorim..

[32] Sujjavanich S., Suwanvitaya P., Chaysuwan D., Heness G. (2017). Synergistic effect of metakaolin and fly ash on properties of concrete. Constr. Build. Mater..

[33] Hassan K., Cabrera J.G., Maliehe R.S. (2013). Effect of mineral admixtures on the properties of high-performance concrete. Cem. Concr. Compos..

[34] Bharatkumar B.H., Narayanan R., Raghuprasad B.K., Ramachandramurthy D.S. (2001). Mix proportioning of high performance concrete. Cem. Concr. Compos..

[35] Nehdi M., Mindess S., Aıtcin P.-C. (1998). Rheology of high-performance concrete: Effect of ultrafine particles. Cem. Concr. Res..

[36] Feng J., Liu S., Wang Z. (2015). Effects of ultrafine fly ash on the properties of high-strength concrete. J. Therm. Anal. Calorim..

[37] Koh K.T., Ryu G.S., Park J.J., An K.H., Kim S.W., Kang S.T. Effects of the composing materials on the rheological and mechanical properties of ultrahigh performance concrete (UHPC). Proceedings of the RILEM-fib-AFGC International Symposium on UHPFRC.

[38] Jiao D., Shi C., Yuan Q., An X., Liu Y., Li H. (2017). Effect of constituents on rheological properties of fresh concrete—A review. Cem. Concr. Compos..

[39] Park C., Noh M., Park T. (2005). Rheological properties of cementitious materials containing mineral admixtures. Cem. Concr. Res..

[40] Zhang X., Han J. (2000). The effect of ultra-fine admixture on the rheological property of cement past. Cem. Concr. Res..

[41] Cyr M., Legrand C., Mouret M. (2000). Study of the shear thickening effect of superplasticizers on the rheological behaviour of cement pastes containing or not mineral additives. Cem. Concr. Res..

[42] Rajadurai R.S., Kang S.T. (2021). Influence of inter-particle distances on the rheological properties of cementitious suspensions. Materials.

[43] Suzuki M., Oshima T. (1983). Estimation of the coordination number in a multi-component mixture of spheres. Powder Technol..

[44] Xu F., Shi X. (2018). Characteristics and applications of fly ash as a sustainable construction material: A state-of-the-art review. Resour. Conserv. Recycl..

[45] Laskar A.I., Talukdar S. (2008). Rheological behavior of high performance concrete with mineral admixtures and their blending. Constr. Build. Mater..

[46] Bergstrom L. (1997). Hamaker’s constants of inorganic materials. Adv. Colloid Interface Sci..

[47] Ackler H.D., French R.H., Chiang Y.-M. (1996). Comparisons of Hamaker constants for ceramic systems with intervening vacuum or water: From force laws and physical properties. J. Colloid Interface Sci..

[48] De Larrard F. (1999). Concrete Mixture Proportioning.

[49] Coe J.R., Godfrey T.B. (1944). Viscosity of water. J. Appl. Phys..

[50] Struble L., Sun G.-K. (1995). Viscosity of portland cement paste as a function of concentration. Adv. Cem. Based Mater..

[51] Do T.-A.L., Hargreaves J.M., Wolf B., Hort B., Mitchell J.R. (2007). Impact of particle size distribution on rheological and textural properties of chocolate models with reduced fat content. J. Food Sci..

[52] Mueller S., Llewellin J.M., Mader H.M. (2011). The effect of particle shape on suspension viscosity and implications for magmatic flows. Geophys. Res. Lett..

[53] Pabst W., Gregorova E., Bertold C. (2006). Particle shape and suspension rheology of short-fiber systems. J. Eur. Ceram. Soc..

